# The effects of rod and cone loss on the photic regulation of locomotor activity and heart rate

**DOI:** 10.1111/j.1460-9568.2008.06388.x

**Published:** 2008-08

**Authors:** Stewart Thompson, Daniela Lupi, Mark W Hankins, Stuart N Peirson, Russell G Foster

**Affiliations:** Nuffield Laboratory of Ophthalmology, The John Radcliffe Hospital, Headley Way, University of OxfordRoosevelt Drive, Oxford OX3 9DU, UK

**Keywords:** activity, light, melanopsin, mice, photoreceptor

## Abstract

Behavioral responses to light indirectly affect cardiovascular output, but in anesthetized rodents a direct effect of light on heart rate has also been described. Both the basis for this response and the contribution of rods, cones and melanopsin-based photosensitive retinal ganglion cells (pRGCs) remains unknown. To understand how light acutely regulates heart rate we studied responses to light in mice lacking all rod and cone photoreceptors (*rd/rd cl *) along with wild-type controls. Our initial experiments delivered light to anesthetized mice at Zeitgeber time (ZT)16 (4 h after lights off, mid-activity phase) and produced an increase in heart rate in wild-type mice, but not in *rd/rd cl* animals. By contrast, parallel experiments in freely-moving mice demonstrated that light exposure at this time suppressed heart rate and activity in both genotypes. Because of the effects of anesthesia, all subsequent studies were conducted in freely-moving animals. The effects of light were also assessed at ZT6 (mid-rest phase). At this timepoint, wild-type mice showed an irradiance-dependent increase in heart rate and activity. By contrast, *rd/rd cl* mice failed to show any modulation of heart rate or activity, even at very high irradiances. Increases in heart rate preceded increases in locomotor activity and remained elevated when locomotor activity ceased, suggesting that these two responses are at least partially uncoupled. Collectively, our results show an acute and phase-dependent effect of light on cardiovascular output in mice. Surprisingly, this irradiance detection response is dependent upon rod and cone photoreceptors, with no apparent contribution from melanopsin pRGCs.

## Introduction

Environmental irradiance regulates multiple aspects of physiology and behavior that enable an organism to exploit both predictable and stochastic changes in the light environment (Foster, 2002). For example, mice segregate their 24-h activity/rest cycle by synchronizing (entraining) an endogenous circadian clock and by using light to acutely suppress activity (negative masking; Mrosovsky *et al.*, 2001; Foster, 2002). The discovery that some responses to irradiance persist in the absence of the rods and cones (Freedman *et al.*, 1999; Lucas *et al.*, 1999; Mrosovsky *et al.*, 2001) provided the conceptual framework for the discovery of a sub-set of photosensitive retinal ganglion cells (pRGCs) that express the photopigment melanopsin in rats (Provencio *et al.*, 1998; Berson *et al.*, 2002) and mice (Sekaran *et al.*, 2003). It has become clear that responses to environmental irradiance are mediated by both rods/cones and pRGCs. For example, pupil constriction is regulated by the rods/cones at low irradiances, whilst full constriction under bright light is dependent upon melanopsin (Lucas *et al.*, 2003).

Many vertebrates show a modulation of cardiovascular output in response to light, often as a result of altered metabolic demand with changes in activity levels (Tankersley *et al.*, 2002). However, two studies in anesthetized and immobile mice suggest a direct photoreceptive regulation of cardiovascular output (Mutoh *et al.*, 2003; Barnard *et al.*, 2004). These responses to light were absent in anesthetized mice lacking functional rods and cones (*rho*^*−/−*^*cng3A*^*−/−*^), suggesting that the melanopsin pRGCs provide little or no contribution to this task (Barnard *et al.*, 2004). In this regard the modulation of heart rate differs from all other irradiance detection responses characterized to date. However, these results from anesthetized mice may not truly reflect the natural physiology of the animal. For example, both visual pathways and cardiovascular responsiveness can be affected by anesthesia (Baldridge, 1996; Chaves *et al.*, 2001). Furthermore, cardiovascular activity is also regulated to meet the demands of specific behaviors and activity states (Dampney *et al.*, 2002). The imposed immobility produced by anesthesia might also suppress such behavioral components of the light response.

The primary aims of this study were to investigate the modulation of heart rate and locomotor activity by light in unanesthetized animals during the mid-activity and mid-rest phase, and to determine the contribution of rods/cones and melanopsin pRGCs in this response. To this end, we recorded both locomotor activity and heart rate in unrestrained mice using implanted radiotelemetry transmitters. Responses of mice to light were assessed within their home-cage and free from extraneous stimuli. To isolate the photoreceptors contributing to these irradiance detection tasks, responses to light were measured in a mouse model lacking all rod and cone photoreceptors (*rd/rd cl *) and compared with wild-type controls.

## Materials and methods

### Animals

All aspects of animal work were carried out under licence and in accordance with the Animal (Scientific Procedures) Act 1986, UK. Age-matched, adult male C3H/He wild-type and *rd/rd cl* mice were used throughout. The *rd/rd cl* mouse model combines a homozygous retinal degeneration (*rd/rd* or *Pde6b*^*rd1*^) mutation with an attenuated diphtheria toxin-human red cone promoter transgene (*cl*) to provide a complete ablation of rod and cone photoreceptors by 80 days old (Lucas *et al.*, 1999). Although there is no identified rod or cone function in the *rho^−/−^ cng3A^−/−^* mice used previously (Barnard *et al.*, 2004), the photoreceptor cells of the outer retina are still retained in this model. Here we utilize the well-characterized *rd/rd cl* model, where the later loss of rods and cones avoids any developmental reorganization of the light input pathway. All irradiance measurements were in μWcm^2^ with a PM203 power meter (Macam Photometrics, Livingston, UK). Approximate corresponding lux values are shown for comparative purposes. Mice were routinely maintained under a fixed 380 μWcm^2^ (∼1125 lux) 12 : 12 h light : dark (LD) cycle unless stated otherwise. Under these conditions, light onset is designated Zeitgeber time (ZT)0, and dark onset ZT12. For mice under a 12 : 12 h LD cycle, the active phase usually spans ZT12–24.

### Responses to light under anesthesia

The effect of light on heart rate was measured in anesthetized mice according to previously described methods (Barnard *et al.*, 2004). Briefly, C3H/He wild-type (*n*=5) and *rd/rd cl* mice (*n*=4) were anesthetized by subcutaneous injection of urethane (∼1.87 mg/mL) 2–4 h after dark onset (ZT14–16 in a 12 : 12 h LD cycle). Under stable anesthesia, electrodes were connected to record activity at 0.1–300 Hz using an Axon Instruments amplifier system (Union City, CA, USA). Animals with stable electrocardiogram recordings were exposed bilaterally to 5-min halogen source white light pulses at 50 and 186 μWcm^2^ (∼560 lux). A rolling 10-beat average was used to generate heart rate in beats per minute. Baseline heart rate was defined as the mean of the 5 min prior to light exposure. Change in heart rate was measured at the peak of responses in wild-type (8 min after onset of the light pulse). Significance was determined using a two-tailed paired Student’s *t*-test. For this data set, significance was defined by *P*=0.05 alpha level, Bonferroni corrected to 0.0125.

### General methods in unrestrained animals

C3H/He wild-type (*n*=5) and *rd/rd cl* mice (*n*=5) with a preoperative weight > 32 g were surgically fitted with PhysioTel® ETA-F20 radiotelemetry biopotential transmitters (Data Sciences International, St Paul, MN, USA). Aseptic surgical techniques were used throughout. Buprenorphine HCl (National Veterinary Supplies, UK) at 0.1 mg/kg of body weight was administered by subcutaneous injection as a pre-operative analgesic. General anesthesia was induced and maintained by isofluorane/oxygen inhalation. Transmitters were fitted to record heart rate and activity according to manufacturer’s guidelines. The positive terminal was anchored to the surface of the abdomen ∼10 mm right of the xiphoid process, and the negative terminal near the right clavicle/pectoral muscle. Baytril® (enrofloxacin) at 5.0 mg/kg of body weight (Bayer Healthcare) was administered by subcutaneous injection as an antibiotic under anesthesia. Rimadyl® (carprofen) at 5.0 mg/kg of body weight (Pfizer Animal Health) was administered by subcutaneous injection as a post-operative analgesic for the first 3 days following surgery. Mice were allowed to recover for > 14 days before use in experiments.

Animals were isolated from possible confounding external stimuli using a modified system of environment control cabinets (Albrecht & Foster, 2002). To record radiotransmitter output, telemetry receiver plates were integrated with the environment control cabinets. Mice were individually housed in custom cages that confined the animal to a light exposable field (25 × 30 cm), with food and water provided *ad libitum.* These cages were then mounted onto the receiver plates. To prevent electromagnetic interference, the timing and irradiance of light stimuli in the cabinets was provided by a custom overhead fiber-optic and light source system (Schott AG, Mainz, Germany). Irradiance was adjusted by mounting neutral density interference filters (BFiOPTiLAS, Milton Keynes, UK) over the face of the fiber-optic within the SpectraStar150 light source. The fibre-optic cable was sub-divided into 12 strands, with four mounted over each cage to provide even lighting in the cage area.

Heart rate and activity for individual mice was sampled continuously in 2-s bins at 30-s intervals and recorded against a date/time stamp using the dataquest A.R.T.™ software (Data Sciences International). Heart rate as beats per minute was derived from mean cardiac inter-beat (R to R) intervals and assessed statistically as described in each specific protocol. Activity data were recorded in arbitrary lateral movement ‘units’.

### Circadian rhythm measurements

To prescreen animals for normal circadian patterns of activity and heart rate, recordings were first made in wild-type (*n*=5) and *rd/rd cl* mice (*n*=4) maintained in constant darkness. Mice were checked daily using night vision goggles. The mean level and amplitude of locomotor activity and heart rate were assessed over a 3-day period. Comparison between genotypes was by two-tailed unequal variance Student’s *t*-test. For this data set, significance was defined by *P*=0.05 alpha level, Bonferroni corrected to 0.0125.

### Active phase responses (ZT16)

To assess the suppression of locomotor activity by light during the nocturnal active phase (negative masking) in freely-moving animals, wild-type (*n*=3) and *rd/rd cl* mice (*n*=3) mice entrained to a 12 : 12 h LD cycle were exposed to 20-min bright light pulses (380 μWcm^2^, ∼1125 lux) starting approximately 4 h after lights off (ZT16 ± 30 min), in the mid-active phase. The effect of light on activity was described by 5 min of baseline data immediately preceding the light pulse, and the last 5 min of the light pulse. Comparisons were by paired Student’s *t*-test. Comparison of change in activity and heart rate between genotypes was by two-tailed unequal variance Student’s *t*-test. For this data set, significance was defined by *P*=0.05 alpha level, Bonferroni corrected to 0.0125.

In addition, to verify the previously demonstrated capacity to sustain negative masking responses of wild-type and *rd/rd cl* mice, further tests were made in a limited group of animals with radiotelemeters still functional after completion of all planned assays. Wild-type and *rd/rd cl* mice were entrained to a 12 : 12 h LD cycle and exposed to a 60-min 380 μWcm^2^ (∼1125 lux) pulse of light starting at ZT16. These additional tests were successfully completed in wild-type (*n*=1) and *rd/rd cl* (*n*=1) mice. For presentation, baseline heart rate and activity measurements were taken from the 20-min period immediately preceding the light pulse, and activity and heart rate expressed as a percentage of baseline.

### Quiescent phase responses (ZT6)

Mice were entrained to a 12 : 12 h LD cycle as described above. On experimental days the lights were programmed so that mice remained in darkness during the first half of what would normally be the light phase (ZT0–6). Twenty-minute defined irradiance pulses of light were applied at what would have been the mid-light phase (ZT6). At the end of the light pulse, entraining lights were reinstated (380 μWcm^2^). All animals were exposed to 380 μWcm^2^ (∼1125 lux), 40 μWcm^2^ (∼190 lux), 6.0 μWcm^2^ (∼20 lux), 0.75 μWcm^2^ (∼2 lux), 0.075 μWcm^2^ (∼0.2 lux) and 0.0075 μWcm^2^ (∼0.02 lux).

Although light pulses were applied at a phase of the endogenous circadian rhythm when mice are usually at rest, animals do show short bouts of activity. To control for activity, any records showing activity above the first quartile of the maximal activity for that animal, during the 20 min preceding the light pulse were excluded from further analysis. Pulses at all irradiances were replicated for each animal and when repeated measures were available after application of exclusion criteria, the mean of these was used for analysis.

Response magnitude was determined from the 5 min immediately preceding the pulse compared with the 5 min following onset of light exposure, and compared between genotypes by two-tailed unequal variance Student’s *t*-test. The effect of irradiance in wild-type heart rate was tested by repeated-measures anova with a *post hoc* test for a linear trend. The latency for responses was described as the mean interval between pulse onset and the first recording within the upper quartile of the induced response. Differences in the latency of heart rate and activity were compared by two-tailed unequal variance Student’s *t*-test. For this data set, significance was defined by *P*=0.05 alpha level, Bonferroni corrected to 0.0125.

## Results

### Responses to light under anesthesia

**Fig. 1:**
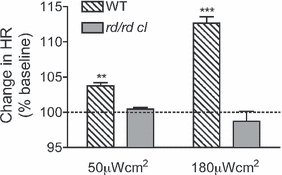
Responses to light under anesthesia. The mean change in heart rate (HR) is shown as a percentage of the corresponding baseline (dotted line at 100%) in wild-type (WT) and *rd/rd cl* mice. Light exposure at ZT16 produced an increase in heart rate in anesthetized wild-type mice, but not in *rd/rd cl* mice. Error bars indicate SEM. **P < 0.01, ***P < 0.001, comparing genotypes.

Under anesthesia, light produced a significant increase in heart rate in wild-type mice at 50 μWcm^2^ (*P*=0.002, *t*=7.1) and 186 μWcm^2^ (*P*<0.001, *t*=11.6; Fig. 1). This response was characterized by a gradual increase in heart rate peaking 8 min after onset of the light pulse. No change in heart rate was apparent in anesthetized *rd/rd cl* mice at 50 μWcm^2^ (*P*=0.18; *t*=1.7) or 186 μWcm^2^ (*P*=0.43; *t*=0.9). These results are consistent with our previously published data in *rho*^*−/−*^*cng3A*^*−/−*^ animals (Barnard *et al.*, 2004).

### Circadian rhythm measurements

**Fig. 2:**
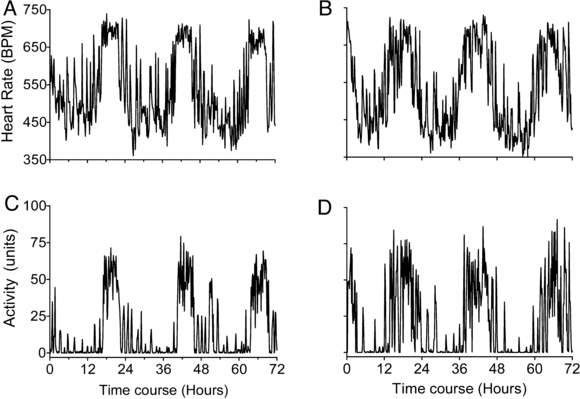
Circadian rhythms of parameters. Daily rhythm in activity and heart rate are shown for a representative (A and C) wild-type and (B and D) *rd/rd cl* mouse over 3 days in constant darkness. Rhythms in parameters were as predicted by previous studies and were not significantly different between genotypes.

Both wild-type and *rd/rd cl* mice showed 24-h rhythms in activity and heart rate that were phase coupled (Fig. 2). There were no significant differences between genotypes in the mean activity (wild-type 10.4 ± 2.30, *rd/rd cl* 11.9 ± 1.15, *P*=0.62, *t*=0.8) or heart rate (mean ± SEM in wild-type 551.2 ± 10.9, *rd/rd cl* 573.8 ± 12.9, *P*=0.22, *t*=1.45). In addition, there were no differences in amplitude of activity (wild-type 78.6 ± 14.3, *rd/rd cl* 75.0 ± 2.2, *P*=0.62, *t*=0.82) or heart rate (mean ± SEM heart rate in wild-type 414.2 ± 24.3, *rd/rd cl* 369.5 ± 7.1, *P*=0.14, *t*=1.8).

### Active phase responses (ZT16)

**Fig. 3:**
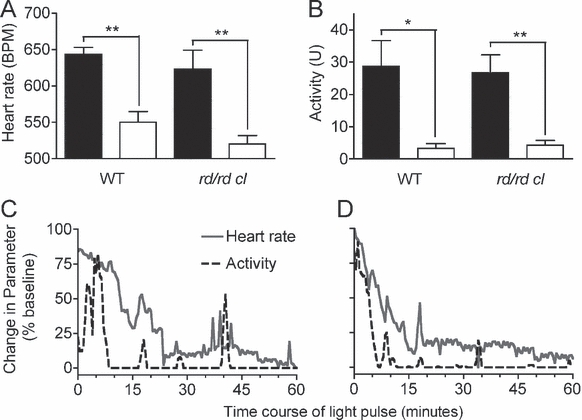
Responses during the active phase (ZT16). Bright light pulses during the active phase produced rapid suppression of activity, followed by a reduction in heart rate, consistent with negative masking by light. Mean and SEM of heart rate (A) and activity (B) are shown for pre-pulse darkness baseline (black filled bars) and during 20-min 380-μWcm^2^ pulses (open bars). Responses were equivalent in wild-type (WT) and *rd/rd cl* mice. The time course of responses in heart rate (gray lines) and activity (black lines) are shown for individual wild-type (C) and *rd/rd cl* mice (D) during a 60-min 380-ìWcm^2^ pulse during the active phase; responses are presented as a percentage of the corresponding baseline. *P < 0.05, **P < 0.01, change with treatment.

Non-anesthetized and freely-moving mice showed a significant suppression of locomotor activity (wild-type *P*=0.011, *t*=9.3, df = 2; *rd/rd cl P*=0.009, *t*=10.2; Fig. 3A and B). Changes in heart rate paralleled changes in activity, and there were no apparent genotype differences of either the change in activity (*P*=0.81, *t*=0.82) or heart rate (*P*=0.96, *t*=0.82). Responses in individual wild-type and *rd/rd cl* mice to a 60-min light pulse verified the capacity to sustain negative masking responses (Fig. 3C and D).

### Quiescent phase responses (ZT6)

**Fig. 4:**
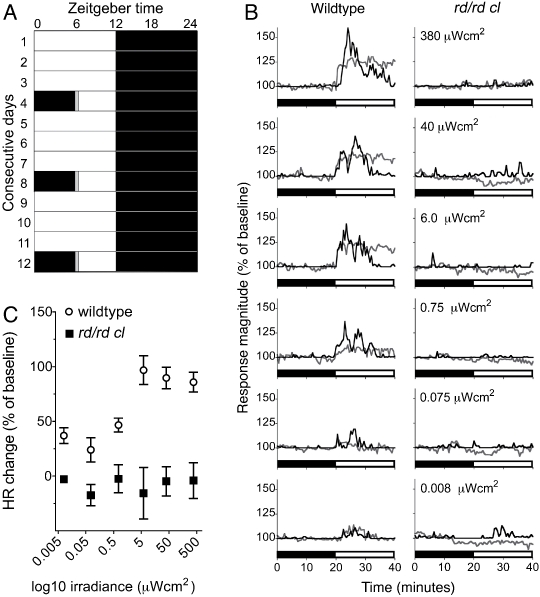
Responses during the quiescent phase (ZT6). (A) The light treatment protocol is shown as the light regime over consecutive days. Shading represents the light condition in the cabinet at a given time on each day. On pulse days the lights do not come on in the morning and a 20-min light pulse is applied in the middle of the quiescent phase before entraining lights are re-instated. (B) The time course shows the 20 min preceding the light pulse and the 20 min of the light pulse, with the dark to light transition shown by a black and white bar. The mean of responses in heart rate (HR; gray lines) and activity (black lines) are presented as a percentage of the corresponding baseline. Irradiance is shown for each pair of panels. An arousal from quiescence, with a simultaneous induction of activity and increase in heart rate was consistently produced in response to dark to light transitions in wild-type mice. In contrast, the *rd/rd cl* mice showed no arousal from quiescence, and no identifiable response in either activity or heart rate to light, even at high irradiances. (C) The data presented in (B) are summarized as a dose (irradiance) response range.

In non-anesthetized wild-type mice, a light pulse at mid-inactive phase (ZT6; Fig. 4A) consistently produced arousal from quiescence with a rapid induction of both activity and heart rate (Fig. 4B). By contrast, *rd/rd cl* mice showed no increase in either activity or heart rate, even at high irradiances (Fig. 4B and C). Statistical comparison between the genotypes shows a significant difference between wild-type and *rd/rd cl* responses to light (*P*=0.003, *t*=5.5). In the wild-type mice the response in heart rate was irradiance dependent (anova*post hoc* test for linear trend *R*^2^ = 0.578, *P*≤0.0001, *t*=9.1).

In terms of the latency from light onset to peak response in wild-type mice, there was no difference between parameters (activity 1.32 min SEM ± 0.17; heart rate 1.15 min SEM ± 0.14; *P*=0.27, *t*=1.5). However, after the initial response, changes in heart rate and activity were not tightly coupled. This was most evident at higher irradiances where heart rate remained elevated beyond the 20 min of the light pulse, whilst locomotor activity returned to quiescence within 10–15 min.

## Discussion

The purpose of this study was twofold. First, to investigate the modulation of heart rate and locomotor activity by light in freely-moving animals during the mid-activity and mid-rest phase. Second, to determine the contribution of rods/cones and pRGCs to these responses.

Similar to previous studies of a *rho*^*−/−*^*cng3A*^*−/−*^ mouse lacking functional rods and cones (Barnard *et al.*, 2004), we observed a transient increase in heart rate in response to light in anesthetized animals at ZT16. This increase was only apparent in the wild-type and did not occur in *rd/rd cl* animals (Fig. 1). This finding strongly contrasted with the effect of light on heart rate in freely-moving animals at ZT16. The reduction in locomotor activity in response to bright light exposure during the active phase, termed negative masking, has been well-characterized (Mrosovsky *et al.*, 2001; Thompson *et al.*, 2008). In this study, we confirmed that negative masking is largely retained in the absence of the rods and cones (*rd/rd cl* mice) and, moreover, that this response is associated with a reduction in heart rate that broadly parallels the reduction in activity (Fig. 3). The decline in heart rate is most simply explained as a consequence of the reduced metabolic demand following the suppression of locomotor activity (Dampney *et al.*, 2002).

Although light has a suppressive effect on activity and heart rate at ZT16, it produced an increase in both locomotor activity and heart rate when delivered to resting wild-type mice in the middle of their subjective day (ZT6; Fig. 4B and C). Light resulted in a rapid increase in locomotor activity and heart rate, consistent with an alerting or defensive response (Keay *et al.*, 1988). Changes in heart rate may be explained as a result of increased activity. However, the light-induced increase in heart rate can be separated from locomotor activity. For example, at saturating light levels increases in heart rate precede the increases in locomotor activity, as shown in Fig. 4B. Moreover, at all irradiances greater than 0.75 μW/cm^2^, increases in heart rate persist beyond the duration of the transient increase in activity, suggesting that heart rate and locomotor activity are at least partially uncoupled. The contribution of locomotor activity to increases in heart rate could be definitively addressed in future studies by studying these responses following the application of neuromuscular blocking agents.

Remarkably, the irradiance-dependent increase in locomotor activity and heart rate during the rest phase (ZT6) did not occur in *rd/rd cl* animals. These data, in the absence of anesthesia, suggest that the rods and cones provide the exclusive input to this irradiance detection task. The finding that pRGCs do not provide any input to the photic modulation of locomotor activity and heart rate is unique among irradiance detection tasks. Melanopsin and rod/cone photoreceptor inputs overlap in circadian entrainment (Hattar *et al.*, 2003; Mrosovsky, 2003), melatonin suppression (Lucas *et al.*, 1999; Rea *et al.*, 2002), negative masking (Mrosovsky & Hattar, 2003) and the pupillary light response (Lucas *et al.*, 2003). It remains unclear what sensory information the different photoreceptors (rods, cones and pRGCs) provide to these processes, but their contribution may be related to both the latency and dynamic range of the response in question. It seems likely that the rods/cones mediate short latency and dim light detection, whilst melanopsin controls longer latency and bright light responses. This hypothesis is consistent with the results presented here, which show that the rapid light-induced rise in heart rate is dependent upon the rods/cones. It is also worth noting that the relative contribution of rods/cones and pRGCs may vary with the time of day, as has been shown in ocular physiology (Barnard *et al.*, 2006).

In summary, we have shown that light independently regulates locomotor activity and heart rate in the mid-rest phase of mice. These responses are dependent upon rod and cone photoreceptors, and in their absence pRGCs cannot compensate for their loss. The anatomical pathways whereby rods and cones influence heart rate remain poorly defined, although both the suprachiasmatic nuclei (Mutoh *et al.*, 2003) and the superior colliculus (Keay *et al.*, 1988) have been implicated in previous studies. Whilst the recently identified melanopsin-expressing pRGCs are justifiably attracting considerable attention, our findings emphasize the need to consider the role of all classes of photoreceptor in irradiance detection tasks.

## Abbreviations

LD: light : dark cycle
pRGC: photosensitive retinal ganglion cell
ZT: Zeitgeber time(relative to an entraining light cycle)
